# A novel microextraction technique aided by air agitation using a natural hydrophobic deep eutectic solvent for the extraction of fluvastatin and empagliflozin from plasma samples: application to pharmacokinetic and drug–drug interaction study†

**DOI:** 10.1039/d3ra05929d

**Published:** 2023-10-24

**Authors:** Khalid Alhazzani, Ahmed Z. Alanazi, Aya M. Mostafa, James Barker, Mohamed M. El-Wekil, Al-Montaser Bellah H. Ali

**Affiliations:** a Department of Pharmacology and Toxicology, College of Pharmacy, King Saud University Riyadh Saudi Arabia; b School of Life Sciences, Pharmacy and Chemistry, Kingston University Kingston-upon-Thames London KT1 2EE UK; c Department of Pharmaceutical Analytical Chemistry, Faculty of Pharmacy, Assiut University Assiut Egypt Almontaser_bellah@aun.edu.eg

## Abstract

This study focuses on the interaction between the antihyperlipidemic drug fluvastatin (FLV) and the antidiabetic drug empagliflozin (EMP), which are commonly co-administered medications. EMP's impact on FLV levels is attributed to its inhibition of organic anion transporting polypeptide 1B1 (OATP1B1), responsible for FLV liver uptake, consequently elevating FLV concentrations in blood. Traditional extraction methods for FLV faced difficulties due to its high hydrophobicity. In this study, a hydrophobic natural deep eutectic solvent (NDES) using air assisted dispersive liquid–liquid microextraction (AA-DLLME) was utilized as an excellent choice for achieving the highest extraction recovery, reaching 96% for FLV and 92% for EMP. The NDES was created through the combination of menthol and hippuric acid in a 4 : 1 ratio, making it a green and cost-effective pathway. Liquid phase microextraction followed by spectrofluorometric measurements of FLV at *λ*_em_ = 395 nm and EMP at *λ*_em_ = 303 nm, with excitation at a single wavelength of 275 nm was carried out. Response surface methodology (RSM) relying on central composite design (CCD) was used to optimize the variables affecting the AA-NDES-DLLME. The optimized conditions for extraction are: NDES volume of 200 μL, centrifugation time of 15 minutes, air-agitation cycle of 6 cycles, and sample pH of 4.0. Under these optimized conditions, the developed method exhibited good linearity and precision. The method showed good recoveries from rabbit plasma samples spiked at varying concentrations of the analyzed compounds. To assess the applicability and effectiveness of the hydrophobic DES, the validated method was applied to extract the studied drugs from rabbit plasma samples after oral administration of FLV alone and in combination with EMP. The pharmacokinetic parameters of FLV were calculated in both cases to investigate any changes and determine the need for dose adjustment.

## Abstract 2

This study focuses on the interaction between the antihyperlipidemic drug fluvastatin (FLV) and the antidiabetic drug empagliflozin (EMP), which are commonly co-administered medications.

## Graphical abstract

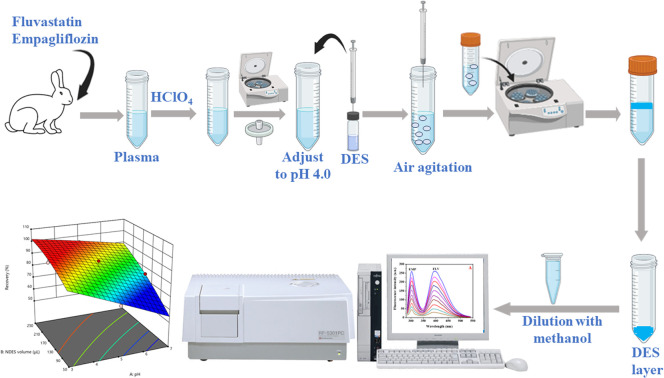

## 1. Introduction

Fluvastatin (FLV) belongs to the class of statin drugs, which is frequently prescribed to lower lipid levels and reduce the risk of cardiovascular disease such as stroke and myocardial infarction.^1^ Empagliflozin (EMP) is a specific sodium-glucose co-transporter 2 (SGLT2) inhibitor that has demonstrated significant benefits among individuals suffering from type 2 diabetes.^2^ The co-administration of SGLT2 inhibitors and statins is a common practice in patients with hyperlipidemia and type 2 diabetes. Recent studies that analyzed randomly collected data revealed that a substantial proportion, up to 77%, of patients who started SGLT2 inhibitor treatment were also prescribed statins.^3^ However, there are previous studies on the interaction between SGLT2 inhibitors and statins that suggest that FLV may interact with EMP.^4–6^ FLV is predominantly taken up by the liver through the action of the organic anion transporting polypeptide 1B1 (OATP1B1), and EMP has been shown to inhibit this transporter.^7–9^ Consequently, the hepatic uptake and clearance of FLV may be reduced, leading to an increase in FLV levels when administered concomitantly with EMP. This interaction should be considered when prescribing EMP in patients who are concurrently taking FLV, and appropriate monitoring and dose adjustments may be necessary to prevent potential adverse effects. The increased FLV levels resulting from its interaction with EMP may lead to several potential side effects such as myopathy,^10^ rhabdomyolysis,^11^ and hepatotoxicity,^12–14^ which may necessitate a reduction in FLV dosage. While the interaction with EMP leading to increased FLV exposure may seem beneficial for enhancing cholesterol reduction, it can also lead to unintended consequences. Even though approved therapeutic doses are used initially, the dramatically elevated FLV levels resulting from the interaction can increase the pharmacological activity beyond intended levels. This suggests extra scrutiny is warranted, as the interaction complicates the pharmacodynamic profile in complex ways. Specifically, the greatly amplified systemic exposure to FLV alters its potency for cholesterol reduction, which may impact both efficacy and safety. Doses that are normally well tolerated could lead to over-treatment and associated adverse effects when FLV levels are multiplied. Furthermore, the already elevated FLV levels due to EMP can be expected to amplify interactions with other inhibitors like CYP3A4 inhibitors, exacerbating the situation. It is therefore evident that close monitoring and possible FLV dose adjustments are necessary to account for the non-linear increases in systemic exposure and associated pharmacological effects. While the enhancement of cholesterol-lowering effect may seem beneficial, the complex pharmacological changes introduce risks like toxicity or overtreatment that warrant careful management.

The combination of fluorescence detection with extraction using hydrophobic deep eutectic solvents (DES) for the analysis of studied drugs offers several advantages over traditional techniques such as high-performance liquid chromatography (HPLC),^15–20^ electrochemical techniques,^21–23^ and spectroscopic methods.^24–29^ Firstly, fluorescence detection provides high sensitivity and selectivity, allowing for the detection and quantification of drugs at low concentrations. Additionally, the use of hydrophobic DES as extraction solvents enhances the extraction efficiency and selectivity for the target drugs, enabling improved sample preparation. This approach also offers the advantage of being relatively simple and cost-effective compared to complex and expensive HPLC systems. Moreover, fluorescence detection combined with hydrophobic DES extraction provides a rapid analysis, saving time when compared to electrochemical techniques or spectroscopic methods.

Dispersive liquid–liquid microextraction (DLLME) is an extraction technique employed for extracting and concentrating analytes from water-based samples. DLLME offers simplicity, rapidity, affordability, high extraction efficiency, and the capability to handle small sample volumes.^30^ Though conventional DLLME uses volatile organic solvents as the extraction media, these solvents suffer from issues like toxicity, volatility, flammability, and negative environmental impact.^31^ So, using a novel type of solvents called deep eutectic solvents (DESs) serve as alternative extractant solvents in DLLME.^32,33^ DESs are a class of green solvents formed from the mixture of a hydrogen bond acceptor (HBA) and a hydrogen bond donor (HBD).^34^ DESs possess characteristics that render them appealing as environmentally friendly alternatives to traditional organic solvents. DESs exhibit characteristics such as low volatility, toxicity, and flammability, making them a favorable option.^34^ Moreover, DESs are cost-effective, simple to prepare, and biodegradable.^35^ Importantly, their properties can be easily tuned by selecting different HBA and HBD components, allowing for customized DESs for specific applications.^36^ The advantages of DESs over conventional solvents include their sustainability due to being prepared from renewable materials, low environmental impact, low costs, variable viscosity, excellent thermal stability and ease of preparation.^34^ DESs have shown good efficacy in extracting a range of analytes such as organic compounds, metals and biomolecules.^36^ When natural terpenes, organic acids, amino acids, sugars, or metabolites are parts of the composition of a deep eutectic solvent (DES), it is known as a natural deep eutectic solvent (NDES).^37^ NDESs, particularly those based on terpenes, closely adhere to the principles of green chemistry.^38^ In 2015, the concept of terpene-based NDESs was initially introduced, combining menthol with organic acids.^39^ These solvents possess both low viscosity and hydrophobic properties, enabling their utilization in situations requiring direct contact with water.^40^ Subsequently, researchers have developed hydrophobic NDES systems using terpenes and other naturally renewable substances. Lately, terpene-derived NDESs have found practical applications in extracting diverse analytes from a range of sources. These include the extraction of different compounds from water and food samples.^41,42^ Moreover, DES-driven methodologies have been successfully employed for the analysis of biological specimens, particularly plasma, serum, and urine.^43–45^ To improve the efficiency of the microextraction procedure, air was introduced as a component. The Air-Assisted Dispersive Liquid–Liquid Microextraction (AA-DLLME) method offers notable advantages in terms of its simplicity, eco-friendliness, cost-effectiveness, and time efficiency when compared to traditional DLLME techniques.^46^

In this work, the synthesized NDES composed of menthol; a monoterpenoid alcohol found naturally in peppermint and other mint plants^47^ as HBA and hippuric acid; key metabolite involved in the detoxification and elimination of aromatic compounds from the body^48^ as HBD at 4 : 1 ratio (Fig. S1†). This NDES was used as an extracting agent to isolate and concentrate FLV and EMP from plasma samples. The aim of this study is to establish an environmentally sustainable and green sample preparation technique using hydrophobic natural deep eutectic solvents (NDESs) for extracting FLV and EMP from plasma samples. To optimize the extraction efficiency, the impact of various crucial factors was investigated using a multivariate approach, specifically the Central Composite Design (CCD) coupled with response surface methodology (RSM).^49^ The extracted drugs in NDES were simultaneously determined based on measuring their native fluorescence at different emission wavelengths after excitation at 275 nm. Fluorometric measurement offers notable advantages due to its excellent sensitivity and selectivity, enabling the detection of even trace concentrations of the studied drugs within plasma samples. It is a fast and straightforward technique, requiring minimal sample preparation, making it cost-effective, time-efficient and not requiring skilled personnel if compared to HPLC.^50,51^ Furthermore, the use of NDES as an extraction medium can enhance the solubility of drugs, improving their extraction efficiency. To evaluate the influence of EMP on FLV levels, a pharmacokinetic study was conducted using rabbits. The FLV levels were carefully monitored both before and after the coadministration of EMP in rabbit plasma. The objective was to evaluate various pharmacokinetic parameters of FLV to determine how EMP influenced the levels of FLV. By analyzing these parameters, we aimed to obtain a deeper understanding into the potential effects of EMP on the pharmacokinetics of FLV.

## 2. Experimental

### 2.1. Chemicals and reagents

Fluvastatin (99.0%) was retrieved from Novartis Pharmaceuticals (Cairo, Egypt). Empagliflozin (99.0%) was gifted by Hikma Pharmaceuticals (Cairo, Egypt). Lescol® capsules containing 40.0 mg FLV per capsule and Faglozino® tablets containing 25.0 mg EMP per tablet were obtained from local drug store. Hippuric acid was purchased from Fluka Analytical (New Jersey, USA). Menthol, thymol, acetonitrile, and methanol were procured from Sigma-Aldrich (Steinheim, Germany). Double distilled water (DDW) was employed throughout experiments.

### 2.2. Instrumentation

Fluorescence emission spectra were obtained from a Shimadzu RF-5301PC fluorescence spectrometer using a 5 nm slit width and installed with a, 1 cm quartz cell (Tokyo, Japan). The centrifugation was carried out using a laboratory centrifuge model 800 (Republic of China). A 100 μL syringe was utilized from Hamilton (USA). A pH-meter device model HI 5222 Hanna (Portugal) and Sartorius Handy balance H51 (Hanover, Germany) were used. Fourier-transform infrared (FT-IR) spectra were obtained using KBr disc on Nicolet 6700 FT-IR-spectrometer (Thermo Electron Corporation, USA) ranging from 400–4000 cm^−1^.

### 2.3. Synthesis of NDESs

The hydrophobic NDES was synthesized by mixing menthol as HBA with hippuric acid as HBD at 4 : 1 ratio in a glass test tube and heated at 80 °C for 40 min until a homogenous clear liquid was obtained. After cooling, the mixture was utilized in the microextraction procedure. Other NDES were prepared *via* a similar procedure with different ratios. The composition of the synthesized NDES and their molar ratios are summarized in Table S1.†

### 2.4. Preparation of calibration standards

Calibration samples were prepared by adding adequate quantities of the stock solution of FLV (10.0 μg mL^−1^) and EMP (10.0 μg mL^−1^) to drug-free rabbit plasma. The final concentrations of the calibration samples after extraction and dilution were from 20.0 to 380.0 ng mL^− 1^ for FLV and from 5.0 to 300.0 ng mL^− 1^ for EMP. The spiked plasma samples were collected and stored in tightly sealed, dark containers at −20 °C. The plasma samples were allowed to thaw at room temperature before analysis.

### 2.5. Plasma samples preparation

Drug-free rabbit plasma was collected from male rabbits that will be involved in the pharmacokinetic study. To prepare the spiked samples, 1 mL of plasma were mixed with 100 μL of standard solutions of FLV and EMP to achieve the desired concentrations. The proteins in the plasma were then removed through precipitation utilizing 7% v/v perchloric acid (HClO_4_), followed by centrifugation at 5000 rpm for 10 minutes. Afterward, the obtained supernatant was separated and passed through a 0.45 μm filter. To minimize the influence of the plasma matrix, the plasma samples were diluted with DDW (double distilled water) till the final volume reach 10.0 mL. The pH of the acidified solution was adjusted to pH 4.0 using the minimum amount of 1 mM NaOH solution. Finally, the drugs were extracted using the prepared NDESs.

### 2.6. Procedure for AA-NDES-DLLME

**Fig. 1:**
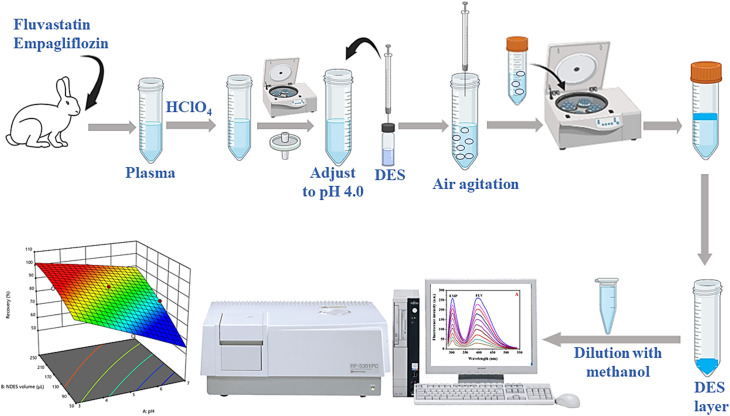
Schematic representation of the AA-NDES-DLLME procedure.

Initially, a 10 mL supernatant of diluted plasma at pH 4.0, which was spiked with a standard solution of FLV and EMP, was loaded into a 15 mL centrifuge tube. Next, 200.0 μL of a hydrophobic DES composed of menthol and hippuric acid in a 4 : 1 ratio was promptly injected into the aqueous solution using a glass microsyringe. To enhance the mass transfer of the drugs to the DES phase, the mixture was repeatedly pushed in and pushed out of the tube six times with the glass syringe, breaking down the aggregated NDES droplets into smaller ones. Afterward, the mixture was subjected to centrifugation at 6000 rpm for 15 minutes, leading to the separation of the hydrophobic NDES-rich phase from the aqueous medium. Because of the reduced density of the NDES phase in comparison to water, the DES-enriched phase was gathered at the upper portion of the solution, while the lower aqueous phase was extracted using a syringe. The hydrophobic NDES phase was carefully gathered from the tapered segment of the tube using a microsyringe. Finally, the volume was brought to 1.0 mL by addition of methanol, and the fluorescence intensity of FLV and EMP was measured at 303 nm and 395 nm, respectively, following excitation at 275 nm. The schematic representation of the AA-NDES-DLLME procedure can be shown in Fig. 1.

Subsequently, the effectiveness of the proposed procedure was investigated by determining the percent extraction recovery (% ER) and the enrichment factor (EF).
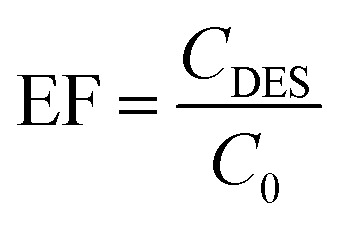

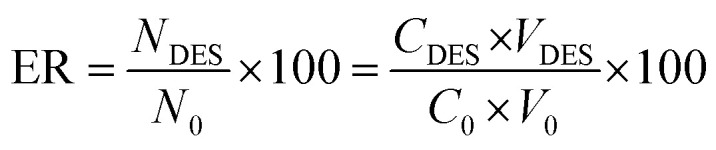
where *C*_DES_ and *C*_0_ are the final and starting concentrations of the studied drugs in DES layer and the original solution containing sample, respectively; *N*_DES_ and *N*_0_ are the final and initial number of moles found in the DES layer and sample solution, respectively; *V*_0_ is the volume of the sample solution; and *V*_DES_ is the volume of the DES phase.

### 2.7. Experimental design methodology

To evaluate the effects of different parameters and refine the AA-NDES-DLLME procedure for the drugs under investigation, an experimental design method is employed. This method presents an advantage in comparison to the classical approach of optimizing one variable at a time by providing a more comprehensive understanding of the interaction of variables on the extraction process. In this study, the central composite design (CCD) in conjunction with the response surface methodology (RSM) was utilized to optimize the influential factors. The design consisted of 1 block, 5 levels, and 4 factors, resulting in the lowest possible trials, which included 16 fractional factorial points, 8 axial points, and 6 central points, representing +1 (or −1), +*α* (or −*α*), and zero levels, respectively. The main experimental factors affecting the microextraction efficiency were identified through preliminary tests and included sample pH (ranging from 3.0 to 7.0), DES volume (ranging from 50.0 to 250.0 μL), centrifugation time (ranging from 5.0 to 25.0 minutes), and the number of air-agitation cycles (ranging from 2 to 10). Table S2† presents the operational levels for the four factors, along with the design matrix and experimental responses in terms of extraction recovery. Optimal conditions for the four variables and their interactions were determined. A significance level of less than 5% (*p* < 0.05, *t*-test) and a lack of fit level with *p* > 0.05 (*F*-test) were used for all statistical analyses. The experimental design was conducted using the Design Expert software (Version 13, Stat-Ease Inc., USA).

### 2.8. Animals

Ethical approval (under the No. 06/2023/0111) for this research was granted by the ethics committee at Assiut University, Egypt, and the study was conducted in adherence to the principles outlined in the Declaration of Helsinki. The study involved six New Zealand white rabbits, each weighing 2.0 ± 0.2 kg, obtained from a licensed animal supplier in Assiut, Egypt. Before commencing the study, the rabbits' health was evaluated, ensuring they were in good condition. The rabbits were individually housed in cages measuring 0.8 × 0.6 × 0.5 m and given a 15 day acclimatization period to adjust to the experimental conditions and handling. During this time, no medications or vaccines were administered to the rabbits. The room temperature was kept constant at 25 ± 2 °C, and the relative humidity was maintained at 50 ± 5%. Throughout the acclimatization period, the rabbits were provided with a commercial pellet diet and water. Before the experiment began, the rabbits fasted for approximately 12 hours while being allowed access to water.

### 2.9. Pharmacokinetic application

The rabbits administered FLV *via* oral route at a dose of 2.0 mg kg^−1^. After a washout period of 14 days to allow any FLV to clear from the rabbits' bodies, the same rabbits administered both (2 mg kg^−1^ for FLV and 1.5 mg kg^−1^ for EMP). Blood samples (1.0 mL) were collected after administration of the studied analytes after 0.25, 0.5, 1, 1.5, 3, 5, 7, 10, 12, 18 and 24 h of their administration. The samples were drawn from marginal ear vein, placed into tubes that had been heparinized, and centrifuged for 15 minutes at 6000 rpm. The plasma was then collected and stored at −20 °C in dark containers until analysis. The pharmacokinetic parameters of FLV were calculated using one compartment model for the treated group using Phoenix®WinNonlin software version 5.1 (Pharsight, Mountain View, CA, USA). These parameters included total area under the curve (AUC) from 0 to 24 hours (AUC_0→24_), cumulative plasma concentration from 0 to infinity (AUC_0→∞_), peak plasma concentration (*C*_max_), elimination rate constant (*K*_e_), mean residence time (MRT), absorption half-life (*t*_1/2a_), and elimination half-life (*t*_1/2_). The data are reported as mean ± standard deviation (mean ± SD). To analyze differences in parameters between the groups, *t*-test was employed, using GraphPad Prism 9.0. A *p*-value < 0.05 was found to be statistically significant, indicating differences between paired data sets.

## 3. Results and discussion

### 3.1. Characterization of DESs

**Fig. 2:**
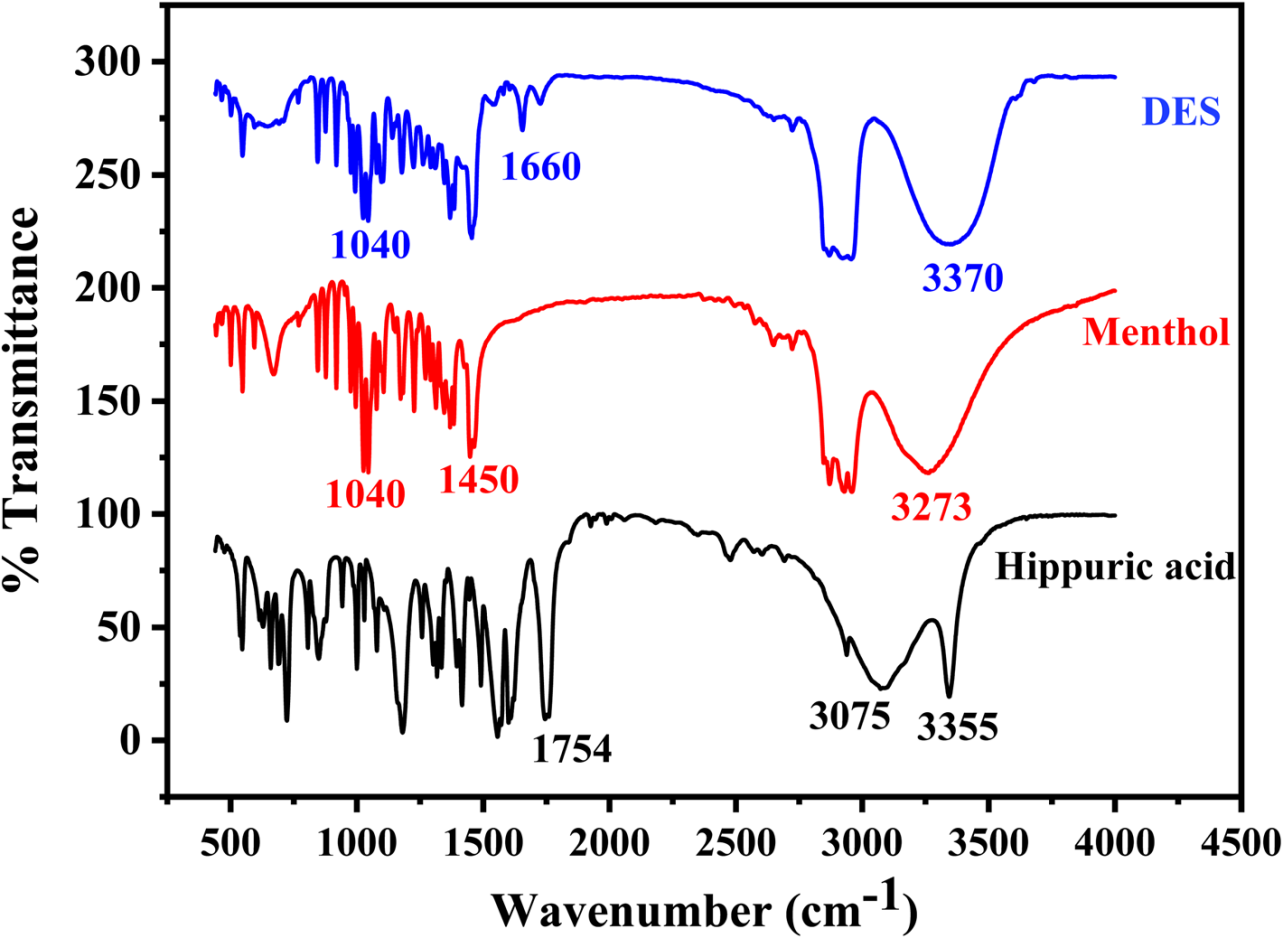
FT-IR spectra of hippuric acid as HBD, menthol as HBA, and DES (hippuric acid : menthol) synthesized in molar ratio 1 : 4.

The FT-IR spectrum of NDES resulting from the interaction between menthol and hippuric acid reveals significant changes in comparison to the individual spectra of the two compounds (Fig. 2). The FT-IR spectra of the individual components display characteristic signals corresponding to their respective functional groups. For menthol, a broad O–H stretching band is detectable at 3273 cm^−1^, C–O stretch appears at 1040 cm^−1^, along with a sharp O–H bend at 1450 cm^−1^.^52^ In the spectrum of hippuric acid, an intense sharp N–H stretching peak is seen at 3355 cm^−1^ assigned to the –NH group. A broadened peak with maximum at 3075 cm^−1^ corresponds to hydrogen-bonded O–H stretches of carboxylic acid. An intense, sharp C=O stretching peak from the carboxylic acid carbonyl occurs at 1754 cm^−1^. Aromatic ring vibrations and C–N stretches are located between 1604–1494 cm^−1^.^53^ For the DES formed from menthol and hippuric acid, several noticeable spectral changes are observed. A single broader O–H stretch appears at 3370 cm^−1^ with a shift to higher wavenumber, while the N–H stretch of hippuric acid at 3355 cm^−1^ disappears or combined with O–H band. The original sharp C=O stretch at 1754 cm^−1^ shifts down to 1660 cm^−1^ and decreases in intensity. The C–O stretch of menthol around 1040 cm^−1^ also becomes less intense. Lastly, the aromatic and C–N vibrations of hippuric acid between 1604–1494 cm^−1^ significantly diminish. These spectral changes suggest hydrogen bond formation between the functional groups of the two components in the deep eutectic mixture. The shifts and intensity reductions in the N–H, O–H, C=O, and C–O bands provide evidence for intermolecular hydrogen bonding interactions involving the hippuric acid amide, carboxylic acid, and menthol hydroxyl groups. Disruption of the hippuric acid aromatic ring is also apparent from the near disappearance of C=C and C–N signals.^39^ In summary, analysis of the FT-IR spectra implies hydrogen bonding drives the interactions in the deep eutectic solvent formed from menthol and hippuric acid.

### 3.2. Selection of extraction solvent

Selecting the right extraction solvent is a critical determinant in the extraction procedure. When using DESs as extraction solvents, it is important that they exhibit specific characteristics. These include high hydrophobicity, effective interaction with the analyte, low solubility in the aqueous solution, dispersibility in the aqueous phase, and stability in the presence of water. The properties of DESs can be controlled by varying the types and proportions of their components, allowing for selective extraction of the target analytes. Three DESs were prepared, each with a different composition ratio, to selectively extract FLV and EMP (Table S1†). The influence of the produced DESs on the recovery of FLV and EMP at different molar ratios (1 : 1, 1 : 3, 1 : 4) was investigated. The obtained results indicated that the DES from hippuric acid : menthol (1 : 4) showed the highest recovery if compared to the other DESs. The NDES formed from menthol and hippuric acid contains both hydrophobic and hydrophilic regions that can interact with FLV and EMP through different mechanisms. FLV consists of a hydrophobic fluorinated phenyl group and a hydrophilic carboxylic acid group; however, the hydrophobic nature prevails, as indicated by the log *P* value of 4.5.^54^ This highlights that choosing a hydrophobic DES is a good option for the extraction process. The cyclic and nonpolar structures of menthol and hippuric acid enable hydrophobic interactions and π–π stacking with the aromatic groups of the drugs, allowing good solubility of the hydrophobic portions. Additionally, the carboxylic acid group of hippuric acid can participate in hydrogen bonding with the carboxylic acid moieties on FLV and EMP. This provides a hydrophilic interaction site to improve solubility of the polar regions of the drugs. This allows the DES to solubilize FLV. EMP also contains both hydrophobic aromatic rings and more hydrophilic hydroxyl groups (log *P* 1.7).^55^ Similar to FLV, the dual nature of the prepared DES provides regions for both hydrophobic and hydrophilic interactions to extract EMP. The tailored interactions and solvation effects provided by the menthol and hippuric acid allow the DES to effectively extract and solubilize both the hydrophobic and hydrophilic portions of FLV and EMP. This results in good recovery of both compounds from the extraction into the DES. Superior extraction recovery of FLV is attributed to its high hydrophobicity, aligns well with the use of the prepared hydrophobic NDES to achieve optimal extraction results.

### 3.3. Optimization of important parameters by CCD

Utilizing the Central Composite Design (CCD) as a powerful optimization approach, we examined the interrelationships among factors, encompassing DES volume, sample pH, centrifugation time, and the number of air agitation cycles, along with their influence on extraction recovery (response). Employing the prescribed levels outlined in Table S2,† we conducted experiments focusing on the designated experimental factors, and the resulting the extraction recovery of both analytes are depicted in Table S3.† The effect of the selected parameters was assessed using analysis of variance (ANOVA), and the ANOVA results are summarized in Table 1. The quadratic models for FLV and EMP demonstrated the best performance in modeling the effect of the independent variables. This was confirmed by the low *p*-value of <0.0001, as well as the *F*-value of 0.5964 and 0.2090 for FLV and EMP, respectively, indicating the significance of the model. The *p*-values being below 0.05 and the large *F*-values which is higher than 0.05 which suggest that the proposed quadratic models accurately describe the relationship between the factors and the response at a 95% confidence level.

**Table 1:** Results of ANOVA for CCD design

Source	FLV			EMP		
	Mean square	*F*-Value	*p*-Value	Mean square	*F*-Value	*p*-Value
Model	129.16	37.86	<0.0001	61.10	21.78	<0.0001
*A*-pH	770.67	225.93	<0.0001	2.67	0.9505	0.3451
*B*-NDES volume	888.17	260.37	<0.0001	384.00	136.87	<0.0001
*C*-Centrifugation time	28.17	8.26	0.0116	266.67	95.05	<0.0001
*D*-Air-agitation cycles	10.67	3.13	0.0973	60.17	21.45	0.0003
*AB*	25.00	7.33	0.0162	0.2500	0.0891	0.7694
*AC*	0.2500	0.0733	0.7903	2.25	0.8020	0.3846
*AD*	0.0000	0.0000	1.0000	2.25	0.8020	0.3846
*BC*	0.2500	0.0733	0.7903	9.00	3.21	0.0935
*BD*	1.0000	0.2932	0.5962	16.00	5.70	0.0305
*CD*	2.25	0.6596	0.4294	9.00	3.21	0.0935
*A* ^2^	0.2976	0.0872	0.7718	0.0476	0.0170	0.8981
*B* ^2^	14.58	4.28	0.0564	4.76	1.70	0.2123
*C* ^2^	10.01	2.94	0.1073	23.05	8.21	0.0118
*D* ^2^	70.58	20.69	0.0004	88.05	31.38	<0.0001
Residual	3.41					
Lack of fit	2.78	0.5964	0.7725	2.13	0.2090	
Pure error	4.67					

The final predictive quadratic equations generated for FLV and EMP recovery in terms of the actual experimental factors are shown below:

For FLV:*Y* = 86.67 − 5.67*A* + 6.08*B* + 1.08*C* − 0.67*D* + 1.25*AB* + 0.125*AC* + 0.00*AD* − 0.13*BC* − 0.25*BD* + 0.38*CD* − 0.10*A*^2^ − 0.73*B*^2^ − 0.60*C*^2^ − 1.60*D*^2^

For EMP:*Y* = 85.00 + 0.33*A* + 4.00*B* + 3.33*C* − 1.58*D* − 0.125*AB* − 0.38*AC* − 0.38*AD* − 0.75*BC* + 1.00*BD* + 0.75*CD* − 0.042*A*^2^ − 0.42*B*^2^ − 0.92*C*^2^ − 1.79*D*^2^

The extraction responses (% recovery) were represented by the variable *Y*, while *A*, *B*, *C*, and *D* corresponded to pH, NDES volume, centrifugation time, and the number of air agitation cycles, respectively.

**Fig. 3:**
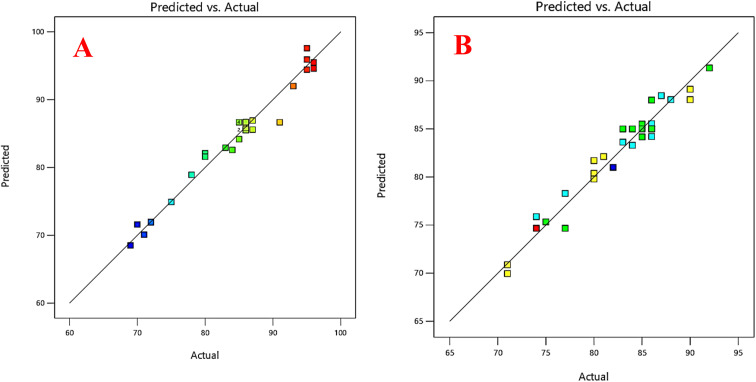
Relationship between the experimental data and the predicted data of the CCD for (A) FLV, (B) EMP.

The developed prediction model showed high accuracy and a good relationship between the experimental data and the fitted model for both FLV and EMP (Fig. 3A and B), with determination coefficients (*R*^2^) of 0.97 and 0.95, and adjusted *R*^2^ values of 0.95 and 0.91, respectively (Table S4†). These coefficients indicate that a significant portion of the variability in the response (extraction % recovery) can be explained by the model. This finding reinforces the reliability of the model and its ability to accurately predict the extraction % recovery for both FLV and EMP. Furthermore, in the resulting model, the variables *A*, *B*, *C*, *D*, *AB*, and *D*^2^ exhibited *p*-values less than 0.05, indicating their statistically significant impact on the efficiency of the suggested FLV extraction approach. Similarly, for EMP, the variables *A*, *C*, *D*, *AB*, *BD*, *C*^2^, and *D*^2^ demonstrated *p*-values below 0.05, confirming their significant effect on the efficiency of the proposed extraction method. These factors played a crucial role in predicting the outcomes of the extraction process. Based on the regression coefficients of the models and the ANOVA, it can be concluded that pH showed a significant negative effect (*p* < 0.05) on extraction recovery of FLV while its effect on extraction recovery of EMP is insignificant (*p* > 0.05). The differing effect of pH on the extraction efficiency of FLV compared to EMP into the hydrophobic NDES can be explained based on their structures. FLV contains a carboxylic acid functional group with a p*K*_a_ around 4.8. At pH values below its p*K*_a_, FLV exists predominantly in its neutral, un-ionized form. However, at pH above 4.8, the carboxylic acid becomes increasingly deprotonated and negatively charged. The hydrophobic NDES would favor partitioning of the neutral, nonpolar form of FLV. As pH increases, more ionized and hydrophilic FLV is present, reducing its solubility in the hydrophobic solvent. This results in decreasing FLV extraction recovery at higher pH. In contrast, EMP does not contain any ionizable groups and it remains essentially neutral over the investigated pH range. Therefore, changes in pH do not significantly alter the hydrophobic–hydrophilic balance of EMP like they do for FLV. In summary, the differential pH effect on FLV and EMP extraction can be attributed to the ionizable carboxylic acid of FLV, making it pH-dependent, *versus* the pH-independent neutral form of EMP. The volume of DES had a positive significant effect (*p* < 0.05) on extraction recovery of FLV and EMP. Besides, centrifugation time and number of air agitation cycles had a significant positive effect on extraction recovery of EMP but its effect on extraction recovery of FLV was insignificant (*p* > 0.05). Conversely, the remaining variables exhibited *p*-values greater than 0.05, indicating that they lacked statistical significance in relation to the predicted results. Thus, these factors were deemed insignificant in the context of the extraction efficiency based on the model.

### 3.4. Response surface methodology

**Fig. 4:**
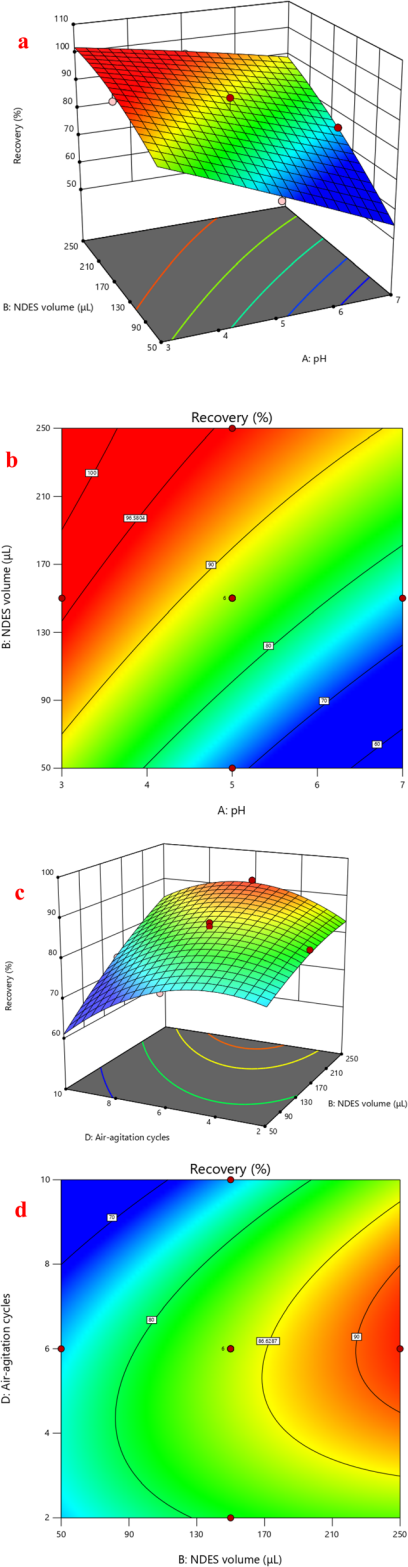
3D-graphs (a) and contour plots (b) of correlation of NDES volume and pH of sample for FLV extraction. 3D-graphs (c) and contour plots (d) of correlation of NDES volume and number of air agitation cycles for EMP extraction.

Response surface analysis was employed to visualize the impact of the optimized factors' binary interactions on the extraction recovery of FLV and EMP. Graphs in three dimensions and the corresponding contour plots were used to study the effect of parameters and their interactions on the extraction recovery which shown in Fig. 4. The surface contour constructed by plotting two variables against each other with the remaining two variables fixed. The interaction of pH and NDES volume had positive significant effects on extraction recovery of FLV (*p* < 0.05). Regarding extraction recovery, increasing volume and decreasing pH gave the highest extraction recovery (Fig. 4A and B). The squared [NDES volume] and squared [air-agitation cycles] interaction having positive effects on the extraction recovery of FLV. The interaction of NDES volume and air-agitation cycles had positive significant effects on extraction recovery of EMP (*p* < 0.05) (Fig. 4C and D). The squared [air-agitation cycles] interaction having negative effects on the extraction recovery of EMP. According to the obtained RSM results, NDES volume of 200 μL, centrifugation time of 15 min, air-agitation cycle of 6 cycles and sample pH of 4 were considered optimal for subsequent experiments.

### 3.5. Optimization of fluorescence detection

A key aspect of developing the analytical fluorescence method was identifying optimal solvent and excitation conditions that provide resolved peaks for both FLV and EMP. After extraction into NDES, the NDES layer was dissolved in different solvents to determine which gave the highest fluorescence intensity. Of the solvents tested, which included methanol, ethanol, and acetonitrile, methanol provided the greatest fluorescence enhancement for both FLV and EMP. Therefore, methanol was selected as the solvent for the extracted DES prior to fluorescence analysis. For excitation wavelength selection, wavelengths ranging from 250–350 nm were evaluated to find a single excitation that could differentiate the two compounds. It was found that excitation at 275 nm allowed both FLV and EMP to fluoresce, but their emissions occurred at distinct resolved wavelengths. Specifically, EMP provided an emission peak at 302 nm, while FLV fluoresced at 395 nm when both compounds were excited at 275 nm. The different emission peaks allowed simultaneous analysis of the two drugs without interference.

### 3.6. Analytical performance of the AA-NDES-DLLME-fluorescence method

In order to verify the effectiveness of the AA-NDES-DLLME-fluorescence approach for extracting and detecting FLV and EMP, various analytical factors were examined under optimized conditions (as detailed in Table S5†). These factors included the linear range (LR), determination coefficient (*R*^2^), limit of detection (LOD) and limit of quantification (LOQ), relative standard deviation (RSD), enrichment factor (EF), and extraction recovery (ER). The validation of the methodology followed the guidelines provided by the International Council on Harmonization (ICH).^56^ The linear range was established through the addition of the investigated drugs at different concentrations, followed by extraction using the proposed procedure. To create a calibration curve, the spiked samples were analyzed in triplicates. The method's linear range for FLV ranged from 20.0 ng mL^−1^ to 380.0 ng mL^−1^, while for EMP, it covers the range of 5.0 ng mL^−1^ to 300.0 ng mL^−1^ (Fig. S2†). The method exhibits an enrichment factor of 48 for FLV and 42 for EMP. The linear equations describing the relationship between concentration (*x*) and response (*y*) are *F* = 0.65*C*_FLV_ + 12.11 for FLV, with a high determination coefficient (*R*^2^) of 0.9986. For EMP, the linear equation is *F* = 0.76*C*_EMP_ + 40.33, with a determination coefficient (*R*^2^) of 0.9966. FLV exhibits an extraction recovery of 96%, whereas EMP demonstrates a recovery of 92%. The method's detection limit (LOD) is 6.3 ng mL^−1^ for FLV and 1.5 ng mL^−1^ for EMP. The quantification limit (LOQ) is 19.2 ng mL^−1^ for FLV and 4.6 ng mL^−1^ for EMP. These limits were determined using formulas: LOD was calculated as 3 times the standard deviation of ten replicate results from the sample blank (*S*_blank_), divided by the slope of the calibration plot (*m*). Similarly, LOQ was calculated as 10 times *S*_blank_ divided by *m*. To assess the precision of the method, standard solutions at three concentrations levels (60, 180, and 300 ng mL^−1^ for FLV, and 30, 110, and 210 ng mL^−1^ for EMP) were analyzed both on the same day and on different days. The results indicated that RSDs fell within the ranges of (1.4–2.7% for FLV, and 2.1–3.2% for EMP) for intra-day precision (with a sample size of 6) and (1.1–2.2% for FLV, and 1.7–2.8% for EMP) for inter-day precision (with a sample size of 18).

### 3.7. Analysis of plasma samples

The suitability of the AA-NDES-DLLME-fluorescence approach to detect FLV and EMP in rabbit plasma samples must be evaluated. Since different species present in the sample could affect how well the method works. The evaluation of matrix effects was carried out using an added-found approach. This involved implementing the proposed method on specific plasma samples that were intentionally spiked with FLV and EMP at three different concentration levels: 60, 180, and 300 ng mL^−1^ for FLV, and 30, 110, and 210 ng mL^−1^ for EMP.

The spiking or relative recovery was determined using the following equation:^57,58^
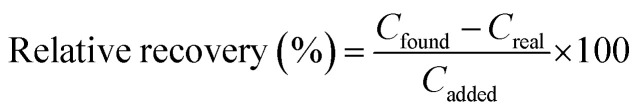
In this context, *C*_found_ represents the concentration of the target analyte detected after adding a certain amount of standard to the real sample. *C*_real_ stands for the initial concentration of the analyte in the actual sample, while *C*_added_ corresponds to the concentration of the specific standard amount that was introduced into the real sample. The results presented in Table S6† reveal favorable average relative recoveries ranging from 95.17% to 96.67% for FLV and from 89.0% to 90.47% for EMP. This confirms that the composition of the sample matrix doesn't notably impact the determination of the analytes. The method demonstrates strong accuracy, possibly due to its effective extracting capability. Furthermore, the RSD% values indicate consistent precision in drug analysis within the plasma sample.

The proposed method was effectively utilized to measure the concentrations of both FLV and EMP in plasma samples obtained from rabbits and the found determination range was (20.0–380 ng mL^−1^ for FLV and 5.0–300 ng mL^−1^ for EMP). The calibration curves for FLV and EMP in spiked rabbit plasma are depicted in Fig. S3.† Notably, the blank plasma sample exhibits minimal interference and a slight background current. This background current is subtracted from the fluorescence signal, resulting in the acquisition of the corrected fluorescence signal. The regression equations that correspond to the obtained results are as follows:*F* = 0.61*C*_FLV_ + 17.64 (*R*^2^ = 0.9966)*F* = 0.77*C*_EMP_ + 45.36 (*R*^2^ = 0.9965)

The limits of detection (LOD) for FLV and EMP were estimated to be 6.82 and 1.71 ng mL^−1^, respectively, whereas the limits of quantification (LOQ) were calculated to be 19.89 ng mL^−1^ and 5.01 ng mL^−1^, respectively.

### 3.8. Selectivity

To ensure the selectivity of measurements in microextraction studies, it is crucial to carefully extract the specific substances of interest from the complex sample mixture. In light of this, the current study extensively explored the capability of the proposed method for analysis of FLV and EMP when applied to spiked rabbit plasma. Under optimized conditions, the selectivity was thoroughly examined against various anions, cations, and other common plasma components. This evaluation involved establishing the maximum allowable concentration of these chemical species that could be present without causing more than a 5% alteration in the analytical signal of FLV or EMP. The findings of this investigation, which are summarized in Table S7,† indicates the selectivity achieved by the optimized methodology for accurately quantifying these target analytes.

### 3.9. Pharmacokinetic application

**Fig. 5:**
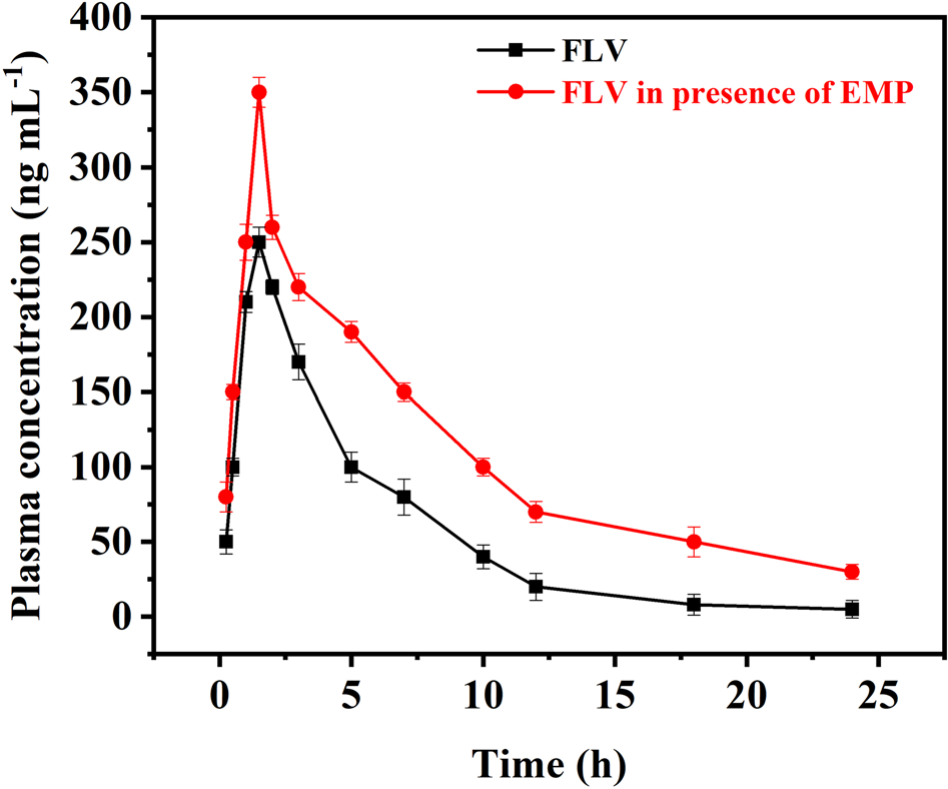
Plasma concentration–time curve after oral administration of FLV and after co-administering FLV with EMP (*n* = 6).

The proposed AA-NDES-DLLME spectrofluorometric method was successfully applied for the quantification of FLV in presence of EMP in rabbit plasma samples for application to pharmacokinetic study in rabbits. Calibration ranges were found suitable to detect samples obtained after co-administration of FLV (2 mg kg^−1^) and EMP (1.5 mg kg^−1^) in rabbits. The highest and lowest plasma levels in samples collected for pharmacokinetic evaluation were found within the calibration range of both drugs. The computed pharmacokinetic parameters are summarized under Table S8.† The observed changes in FLV levels when administered in combination with EMP and impact on pharmacokinetic profile of FLV is shown in Fig. 5. FLV is primarily metabolized by the liver, facilitated by the organic anion transporting polypeptide 1B1 (OATP1B1) transporter. However, EMP has been found to inhibit OATP1B1. This inhibition disrupts the normal hepatic uptake and subsequent clearance of FLV. Consequently, the combination of EMP with FLV can result in altered pharmacokinetic parameters, leading to an increase in FLV levels. When FLV is administered alone, its pharmacokinetic parameters show a certain pattern. The maximum concentration (*C*_max_) achieved is 250.2 ng mL^−1^ at a time (*T*_max_) of 1.5 hours. The elimination rate constant (*K*_e_) indicates a clearance rate of 0.16 h^−1^, with a half-life (*t*_0.5_) of 4.27 h. However, when FLV is co-administered with EMP, the interaction between the two compounds influences FLV's pharmacokinetic profile. The *C*_max_ of FLV increases to 352.2 ng mL^−1^, while the *K*_e_ is reduced to 0.090 h^−1^, elongating the elimination half-life to 7.73 hours. The *K*_a_ and *t*_0.5a_ remain relatively unchanged. These changes are consistent with the inhibition of OATP1B1 by EMP. As EMP inhibits FLV's liver uptake, FLV remains in circulation for a longer duration, leading to higher *C*_max_ and prolonged elimination, as reflected by the increased *K*_e_ and *t*_0.5_ values. This is further confirmed by the increased area under the concentration–time curve for FLV when administered with EMP.

### 3.10. Comparison with other studies

When comparing the proposed AA-NDES-DLLME-fluorescence method with other extraction techniques for FLV and EMP (as shown in Table 2), notable advantages become evident. The attained limits of detection of 1.5 and 6.3 ng mL^−1^ for EMP and FLV are comparable to some reported methods. The wide linear ranges cover therapeutic and toxic concentrations for both drugs in plasma. These analytical ranges meet or exceed those achieved using LC-MS, HPLC, and fluorescence techniques. Recoveries of 92% and 96% for EMP and FLV are higher than or comparable to recoveries of 52–86% from prior solid phase extraction, liquid–liquid extraction, and dispersive liquid–liquid microextraction protocols. This demonstrates the proficiency of NDES-DLLME for efficient extraction of the studied drugs from biological fluids. The eco-friendly, readily preparable NDES replaces hazardous organic solvents like diethyl ether, dichloromethane, methanol and acetonitrile used in existing methods. Combining extraction and quantification in 21 minutes provides faster turnaround *versus* 25–60 minutes for techniques requiring separate optimization steps. Fluorescence detection simplifies quantitation compared to more complex LC-MS-MS instrumentation. Avoiding tandem mass analysis also eliminates extensive method development and validation for each drug. Furthermore, the technique enables cost-effective, simultaneous dual drug analysis without requiring two separate protocols. The analytical efficiency of the AA-NDES-DLLME-fluorescence method confirms its efficacy in extracting FLV and EMP from plasma samples.

**Table 2:** Comparison of the analytical parameters of the optimized AA-NDES-DLLME-fluorescence method with other reported methods for determination of FLV and EMP^a^

Analyte	Analysis technique	Extraction technique	LR (ng mL^−1^)	LOD (ng mL^−1^)	Extraction recovery ± RSD (%)	Total extraction time (min)	Ref.
FLV	LC/MS	SPE Oasis HLB	2–500	2	55.7 ± 4.0	20	59
	HPLC/UV	SPE C_2_ cartridge	0.4–660	0.2	90.0 ± 4.0	29–51	60
	HPLC/fluorescence	LLE	0.0–1000	1.0	94.0 ± 4.9	50	17
	HPLC/UV	SDS	210–29000	190	96.5 ± 1.2	35	61
		TX114 and TBAB	210–16000	140	97.4 ± 1.8	40	
	LC-MS	SPE	NA	0.0037–0.0043	74–86 ± 6.9–10.1	45	15
		DLLME	NA	0.013–0.017	52–68 ± 7.3–13.0	25	
	Fluorescence	NDES	20–380	6.3	96.0 ± 1.9	21	This work
EMP	LC-MS	SPE Oasis MCX	1.50–1500	0.46	87.1 ± 2.3	30	62
	LC-MS	SPE Strata X RP	10.09–403.46	10.09	82.48 ± 2.5	35	20
	Fluorescence	LLE using DEE	500–5000	500	54.61 ± 5.83	60	24
	UHPLC/fluorescence	Quasi-hydrophobic DES	2.0–1.000	0.5	66.0 ± 3.0	15	35
	Fluorescence	NDES	5–300	1.5	92.0 ± 2.5	21	This work

a Solid phase extraction (SPE), dispersive liquid–liquid microextraction (DLLME), nonionic surfactant Triton X-114 (TX-114), tetra-*n*-butylammonium bromide (TBAB), sodium dodecyl sulfate (SDS), mixed-mode cationic exchange (MCX), diethyl ether (DEE), hydrophilic–lipophilic-balanced (HLB), reversed phase (RP).

## 4. Conclusion

In conclusion, this study successfully developed a method for the determination of fluvastatin (FLV) levels in the presence of the antidiabetic medication empagliflozin (EMP). The selective extraction of FLV and EMP was achieved using a hydrophobic natural deep eutectic solvent (NDES), synthesized by combining menthol and hippuric acid in a 4 : 1 ratio. The extraction process employed air assisted deep liquid–liquid microextraction (AA-DLLME), followed by spectrofluorometric measurements of FLV at 395 nm and EMP at 303 nm. The variables influencing the AA-NDES-DLLME steps were optimized using response surface methodology (RSM) based on central composite design (CCD). The optimized conditions included an NDES volume of 200 μL, a centrifugation time of 15 minutes, an air-agitation cycle of 6 cycles, and a sample pH of 4. The developed method offered several advantages, including being environmentally friendly, cost-effective, and demonstrating good linearity, extraction recovery and precision. It provided reliable results for the analysis of FLV and EMP in rabbit plasma samples. Furthermore, the application of the hydrophobic NDES was successfully demonstrated in the extraction of FLV and EMP from rabbit plasma samples after the oral administration of FLV alone and in combination with EMP. The results indicated that the coadministration of EMP led to an increase in FLV levels. This effect can be attributed to the inhibition of organic anion transporting polypeptide 1B1 (OATP1B1) by EMP, which is responsible for the liver uptake of FLV. Consequently, the inhibition of OATP1B1 by EMP resulted in elevated FLV concentrations in the blood. Overall, this study provides valuable insights into the interaction between FLV and EMP and highlights the significance of considering their coadministration in pharmacokinetic studies. The developed method offers a reliable approach for monitoring FLV levels in the presence of EMP, which may have implications for dose adjustment and optimizing therapeutic outcomes.

## Conflicts of interest

The authors declare that they have no known competing financial interests or personal relationships that could have appeared to influence the work reported in this paper.

## Supplementary Material

RA-013-D3RA05929D-s001
