# Supplementary material for: A novel microextraction technique aided by air agitation using a natural hydrophobic deep eutectic solvent for the extraction of fluvastatin and empagliflozin from plasma samples: application to pharmacokinetic and drug–drug interaction study
Source: RSC Adv. 2023 Oct 24;13(44):31201–12. doi: 10.1039/d3ra05929d (PMC10595561; doi:10.1039/d3ra05929d)
Supplement: RA-013-D3RA05929D-s001 [file RA-013-D3RA05929D-s001.pdf]

Electronic Supplementary Material (ESI) for RSC Advances.
This journal is © The Royal Society of Chemistry 2023

Supplementary Data

A novel microextraction technique aided by air agitation using a natural hydrophobic deep eutectic solvent for the extraction of fluvastatin and empagliflozin from plasma samples: Application to pharmacokinetic and drug-drug interaction study

Khalid Alhazzani[a], Ahmed Z. Alanazi[a], Aya M. Mostafa[b,c], James Barker[b], Mohamed M. El-Wekil[c], Al-Montaser Bellah H. Ali[c*]

[a] Department of Pharmacology and Toxicology, College of Pharmacy, King Saud University, Riyadh, Saudi Arabia

[b] School of Life Sciences, Pharmacy, and Chemistry, Kingston University, Kingston-upon-Thames, London KT1 2EE, UK.

[c] Department of Pharmaceutical Analytical Chemistry, Faculty of Pharmacy, Assiut University, Assiut, Egypt.

*e-mail: Almontaser_bellah@aun.edu.eg

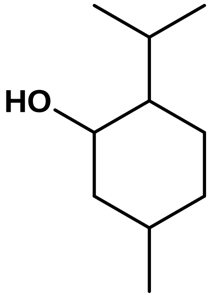

Menthol

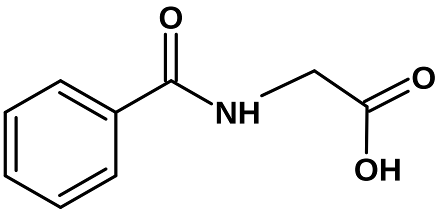

Hippuric acid

4 : 1

Fig. S1. Chemical structure of NDES

Table S1. Preparation of hydrophobic DESs.

HBD	HBA	Mole ratio (HBD: HBA)	Physical state	Extraction recovery (%)	
				FLV	EMP
Hippuric acid	Menthol	1:1	Tubid	-	-
Hippuric acid	Menthol	1:3	Tubid	-	-
Hippuric acid	Menthol	1:4	Clear	96.0	92.0
Hippuric acid	Thymol	1:1	Tubid	-	-
Hippuric acid	Thymol	1:3	Tubid	-	-
Hippuric acid	Thymol	1:4	Tubid	-	-
Butyric acid	Menthol	1:1	Clear	62.0	79.0
Butyric acid	Menthol	1:3	Clear	56.0	84.0
Butyric acid	Menthol	1:4	Clear	51.0	87.0

Table S2. The experimental levels of the factors used in the central composite design.

Factor	Levels of factors				
	Low axial (-α)	Low factorial (-1)	Center (0)	High factorial (+1)	High axial (+α)
**Sample pH**	3	4	5	6	7
**DES volume**	50	100	200	250	250
**Centrifugation time**	5	10	15	20	25
**Number of air-agitation cycles**	2	4	6	8	10

Table S3. CCD matrix and the obtained extraction recoveries.

Run	A: pH	B: NDES volume (μL)	C: Centrifugation time (min.)	D: Air-agitation cycles	Recovery FLV (%)	Recovery EMP (%)
**1**	4	200	20	4	96	88
**2**	5	150	15	6	91	86
**3**	5	50	15	6	70	75
**4**	6	100	20	8	72	80
**5**	6	200	20	4	87	87
**6**	4	100	20	4	87	84
**7**	6	100	20	4	72	86
**8**	6	100	10	4	71	77
**9**	5	250	15	6	95	92
**10**	6	200	20	8	86	90
**11**	5	150	15	2	80	82
**12**	6	100	10	8	69	71
**13**	4	100	10	8	84	71
**14**	6	200	10	4	86	86
**15**	3	150	15	6	95	85
**16**	6	200	10	8	83	81
**17**	4	200	10	4	96	83
**18**	7	150	15	6	75	85
**19**	4	100	10	4	85	74
**20**	5	150	25	6	86	86
**21**	5	150	15	6	85	85
**22**	4	200	20	8	95	90
**23**	5	150	15	6	86	84
**24**	4	100	20	8	86	80
**25**	5	150	15	6	86	86
**26**	4	200	10	8	93	80
**27**	5	150	15	6	86	83
**28**	5	150	15	10	78	74
**29**	5	150	5	6	80	77
**30**	5	150	15	6	86	86

Table S4. Analysis of the variance for the fitted quadratic polynomial model for the extraction of FLV and EMP.

Parameter	FLV	EMP
R^2	0.9725	0.9531
Adjusted R^2	0.9468	0.9093
Predicted R^2	0.8957	0.7684
Adeq Precision	22.2695	18.0473

Table S5. Quantitative parameters of the developed method for the analytes.

Parameter	FLV	EMP
**LR (ng mL^{-1})**	20.0 - 380.0	5.0 - 300.0
**R^2**	0.9986	0.9966
**LOD (ng mL^{-1})**	6.3	1.5
**LOQ (ng mL^{-1})**	19.2	4.6
**EF**	48	42
**ER** (%)	96	92
**Precision, RSD (%)**	Intra-day: 1.4–2.7<br>Inter-day: 1.1–2.2	Intra-day: 2.1–3.2<br>Inter-day: 1.7–2.8

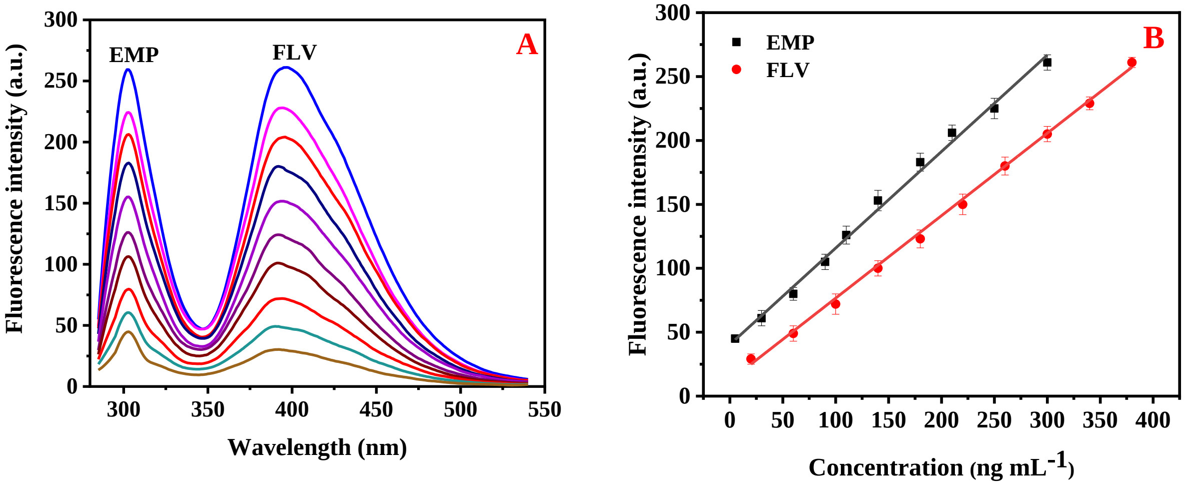

Fig. S2. (A) Fluorescence spectra for different concentrations of FLV and EMP in spiked water samples after NDES-DLLME, (B) the linear calibration curve.

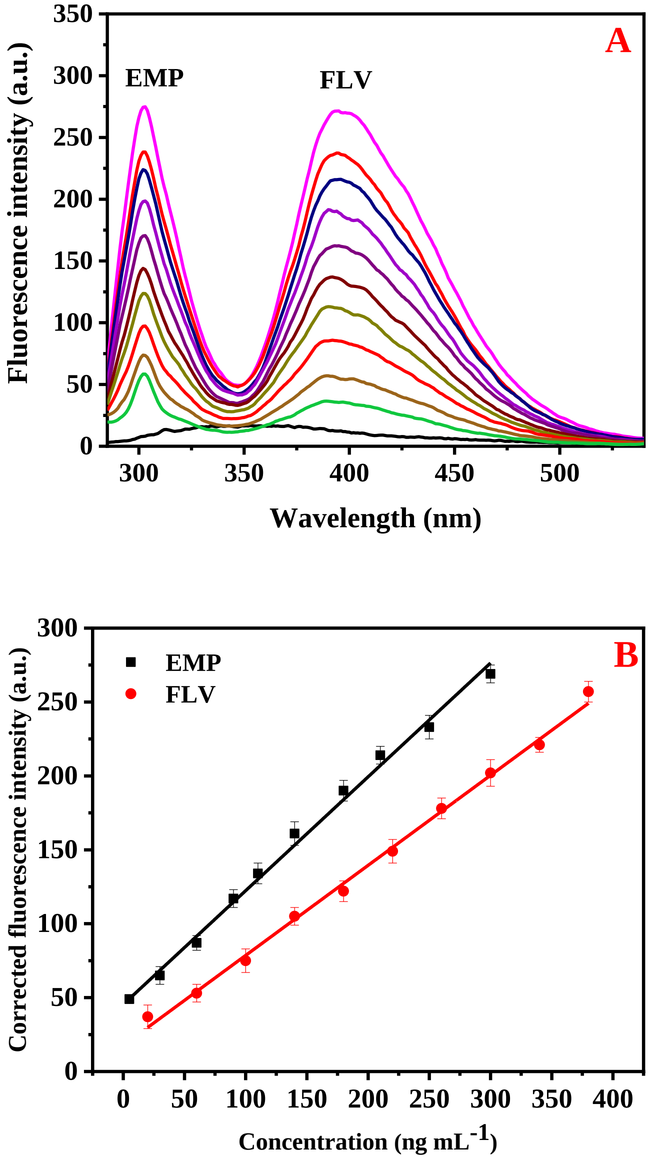

Fig. S3. (A) Fluorescence spectra for different concentrations of FLV and EMP in spiked plasma samples after NDES-DLLME, **Black line** represents blank plasma (B) the linear calibration curves.

Table S6. Relative recoveries (%) from analysis of FLV and EMP in plasma samples at optimum conditions (n=3).

Drug	Amount added (ng mL^{-1})	Amount found (ng mL^{-1})	Relative recovery (RR%)	RSD%
**FLV**	60.0	67.3	95.17	2.12
	180.0	184.2	96.67	1.54
	300.0	299.5	96.43	3.21
**EMP**	30.0	39.5	90.00	2.70
	110.0	109.9	88.54	1.75
	210.0	205.2	91.76	2.81

Amount measured in the real sample (Blank) (at zero concentration of the studied drugs) = 10.2 ng mL^{-1} for EMP and 12.5 ng mL^{-1} for FLV.

Table S7. Selectivity of the proposed method for 100 ng mL^{-1} of FLV and EMP in the presence of different ions and molecules (n = 3).

**Ions and molecules**	FLV	**EMP**
	**Tolerable limit (ng mL^{-1})**	**Tolerable limit (ng mL^{-1})**
Ca^{2+}	3500	4000
K^{+}	5000	3000
SO_4^{2-}	6000	5000
CO_3^{2-}	7000	6000
Mg^{2+}	3000	4000
F^{-}	5000	3000
Cl^{-}	5000	6000
Lactose	1400	1800
Glucose	1800	1900
Zn^{2+}	2500	2600
Fe^{3+}	3500	4500
Tryptophan	700	600
Tyrosine	800	900
Phenylalanine	900	800
Vitamin A	900	700
Riboflavin	600	800
Creatinine	800	900
Pyridoxine	500	1000

Table S8. Pharmacokinetic parameters of FLV after a single oral administration of EMP alone and after oral coadministration of EMP in the studied rabbits (mean ± SD, n = 3)

Parameters	FLV alone	FLV co-administered with EMP	P value*
C_{max} (ng mL^{-1})	250.2 ± 22.58	352.2 ± 20.11	0.0057
T_{max} (hr)	1.5 ± 0.0	1.5 ± 0.0	
K_e (hr^{-1})	0.16 ± 0.024	0.090 ± 0.071	0.0081
$t_{0.5}$ (hr)	4.27 ± 0.78	7.73 ± 0.96	0.0045
K_a (hr^{-1})	2.24 ± 0.42	2.52 ± 0.42	0.058
$t_{0.5a}$ (hr)	0.31 ± 0.14	0.28 ± 0.42	0.079
AUC_{0-24} (ng hr mL^{-1})	1343.0 ± 452.3	2576.25 ± 245.2	0.0012
$AUC_{0-\infty}$ (ng hr mL^{-1})	1367.08 ± 143.5	2896.39 ± 189.25	0.0040
MRT (hr)	6.99 ± 0.89	7.66 ± 0.77	0.059

*A difference was considered significant at $P < 0.05$
